# Power Relations in Optimisation of Therapies and Equity in Access to Antibiotics (PROTEA) Study: investigating the intersection of socio-economic and cultural drivers on antimicrobial resistance (AMR) and its influence on healthcare access and health-providing behaviours in India and South Africa

**DOI:** 10.12688/wellcomeopenres.20193.1

**Published:** 2024-07-24

**Authors:** Esmita Charani, Sipho Dlamini, Anastasia Koch, Sanjeev Singh, Rebecca Hodes, Ramanan Laxminarayan, Deepshikha Batheja, Elelwani Ramugondo, Arunima Sehgal Mukherjee, Marc Mendelson

**Affiliations:** 1 Division of Infectious Diseases and HIV Medicine, Department of Medicine, University of Cape Town, Cape Town, Western Cape, 7925, South Africa; 2 Faculty of Health and Life Sciences, University of Liverpool, Liverpool, UK; 3 Isivivana Centre, Eh!Woza, Khayelitsha, Cape Town, South Africa; 4 Department of Pathology, Faculty of Health Sciences, SAMRC/NHLS/UCT Molecular Mycobacteriology Research Unit& DSI/NRF Centre of Excellence for Biomedical TB Research, Cape Town, South Africa; 5 Institute of Infectious Disease and Molecular Medicine, Faculty of Health Sciences, University of Cape Town, Cape Town, Western Cape, South Africa; 6 Department of Health Sciences Research, Amrita Institute of Medical Sciences, Kochi, Kerala, India; 7 Department of Anthropology, Archaeology and Development Studies, University of Pretoria, Pretoria, South Africa; 8 One Health Trust, Bangalore, India; 9 High Meadows Environmental Institute, Princeton University, Princeton, New Jersey, USA; 10 Department of Rehabilitation Sciences, Faculty of Health Sciences, University of Cape Town, Cape Town, Western Cape, South Africa; 11 Society for Health information Systems Programme (HISP), New Delhi, India; 12 Centre for Sustainable Healthcare Education, University of Oslo, Oslo, Norway

**Keywords:** Intersectionality, Race, Gender, Ethnicity, Infection, Antimicrobial Resistance

## Abstract

Across social structures within society, including healthcare, power relations manifest according to gender, socioeconomic status, race, ethnicity, and class influencing infection related healthcare access and health providing-behaviours. Therefore, accounting for sociocultural drivers, including gender, race, and class, and their influence on economic status can improve healthcare access and health-providing behaviours in infection prevention and control (IPC) as well as antibiotic use, which in turn helps mitigate the spread of antimicrobial resistance (AMR).

This Wellcome funded research will investigate how and why the social determinants of health and economic status influence how people seek, experience, and provide healthcare for suspected or proven (bacterial) infections and how these factors influence antibiotic prescribing and use in South Africa (upper middle-income country) and India (lower middle-income country). The aim of this body of work is to, (1) define and estimate the sociocultural and economic drivers for AMR in different resource settings, (2) design, implement and evaluate context-sensitive IPC and antimicrobial stewardship (AMS) interventions, and (3) inform policy and strategy for AMR mitigation. The population will be healthcare workers (HCWs), patients, and their carers across acute medical and surgical pathways where IPC and antibiotic-related healthcare access and health-providing behaviours will be studied.

Qualitative methods will include ethnographic research, semi-structured in-depth interviews, and focus groups with healthcare providers, patients and carers. Quantitative analysis of bedside observational data from hospitals and population level data on antibiotic use will study the various predictors of AMR using bivariable and multivariable regression analyses. The research will provide high-quality evidence on how social determinants intersect with health, social well-being, and vulnerability in IPC practices and antibiotic use. Using this knowledge we will: 1) design, implement, and measure effects of interventions accounting for these factors; 2) provide a toolkit for advocacy for actors in AMR, and healthcare to assist them to promote dialogue, including policy dialogue on this issue. This work directly benefits the target population and informs healthcare services and practice across the participating countries with potential for wider translation.

The setting will be hospitals in South Africa (middle-income country) and India (lower middle-income country). The population will be healthcare workers (HCWs), patients, and their carers across acute medical and surgical pathways where IPC and antibiotic-related health-seeking and health-providing behaviours will be studied. These populations represent communities most affected by infections and AMR because existing interventions do not address a) differences in how surgical versus medical teams manage infections; b) the role of the wider social network of individuals on their decision-making, c) intersection of the social determinants of health including race, gender, socioeconomic deprivation with AMR.

## Introduction

Bacterial antimicrobial resistance (AMR) is a multifaceted global health challenge, mired by inequity in prioritisation of solutions that match local needs ^1^. The recent Lancet series on AMR has highlighted the significant contribution of basic interventions of water, hygiene and sanitation (WASH), infection prevention, and vaccination in mitigating the threat of AMR in low- and middle-income countries (LMICs) ^2^ and providing achievable targets in delivering change globally ^3^. The drivers and consequences of AMR are socially manifested ^4, 5^. To be sustainable, interventions must account for the structural and social determinants of health ^6– 8^. Healthcare workers (HCWs), patients and their care-givers are vulnerable to risk of infections, and can further help spread pathogens including those that are drug-resistant. To optimise care, we must look at collective and individual behaviours among patients, their carers, and HCWs ^9^. A key priority area for research in AMR recognised in policy, including by the World Health Organization (WHO) is the need to account for and address the behavioural drivers of AMR ^10, 11^.

Socioeconomic and cultural inequities influence healthcare access and health-providing behaviours, impacting morbidity and mortality, as demonstrated during the COVID-19 pandemic ^12^. To mitigate the threat of AMR we need to understand and address these inequities and their consequences ^13^. This study proposes to bridge the gap in understanding by identifying communities, in LMICs, who are most affected by AMR because: 1) their burden of infection is highest, placing them at greatest risk of untreatable infections ^14^; 2) they lack equitable access to programmes for disease prevention - vaccination and WASH ^15, 16^; 3) they are often exposed to conditions that promote the emergence and spread of AMR, including interrupted, inadequate, or inappropriate treatment, and dependence on antibiotics through informal routes without the need for a prescription, and higher rates of access to falsified and substandard antimicrobials ^17^. This will help us understand their exposure to drug-resistant infections from health-providing and healthcare access perspectives based on gender, race and other sources of marginalisation ^4^. These questions are echoed in the WHO report on tackling AMR, which posed the following questions: Is the impact of AMR the same for everyone? Which groups in society face greater or different risks of exposure to AMR or other challenges in accessing, providing, using and benefiting from the information, services and solutions to tackle AMR, including infection prevention and control (IPC)? ^13^ Leaving behind the siloed approaches and considering each inequality and its relationship with AMR, the multitude of social constructs, hierarchies and inequalities, which create axes of power and thereby influence behaviours require renewed focus ^18^.

While power dynamics that impact on people’s health and wellbeing have been described in transdisciplinary literature, solutions to address these imbalances are lacking ^19^. Applying the lens of intersectional inquiry, this research will broaden understandings of the axes of power along which healthcare is provided or denied, and social constructs as predictors of IPC- and AMR-related behaviours. Intersectionality hinges on understanding human beings as shaped by the interaction of different social constructs and conditions (e.g., race, caste, gender identity, class, geography, religion, migration status), which interact within connected systems and structures of power e.g. healthcare systems ^20, 21^. Most of the challenges of inequalities in health are in LMICs, where limited intersectional research has been applied. Recognising these complex interactions and dynamics allows for a better understanding of social inequalities in healthcare access and provision. This will provide a valuable opportunity for researchers, hospital managers, and public health policy-makers to respond to the problems that are identified, optimising the management of AMR among patients as well as the broader public ^22^.

We will use occupational consciousness as an analytical framework to develop a critical tool for self-advocacy and self-awareness to disrupt the existing hierarchies and social norms that govern current practices ^23^. Occupational consciousness describes the ‘awareness about the dynamics of hegemony and recognition that dominant practices are sustained through what people do every day, with implications for personal and collective health’ ^23^. Coined as part of post-colonial research in occupational science, it has applicability well beyond this field. Occupational consciousness helps identify how power intersects with behaviours we observe and provides a language through which people can describe how their individual and collective everyday behaviours and can resist and challenge hegemonic practices that sustain unequal power relations, including in IPC and AMR.

## Rationale

Qualitative research has described professional hierarchies as key determinants of antibiotic decision-making in hospitals ^24– 26^. Using ethnographic research in UK, India and South Africa, we previously investigated differences in antibiotic decision-making and power dynamics between surgical and medical teams, describing how individualistic and hierarchical cultures impact care ^27, 28^. Gendered occupational segregation persists within different roles in the healthcare system ^29, 30^. While 67% of the workforce is female, the majority of physicians are male and the majority of nurses (workforce critical to IPC) are female. Globally, pharmacists and nurses remain excluded from antimicrobial stewardship (AMS) and IPC efforts ^31, 32^. Existing research has demonstrated how gender composition of teams impacts behaviours and experiences of HCWs and patients, leading to gendered leadership models on ward rounds affecting outcomes ^27, 28^. For example, there are more task-driven consultants (predominantly female in our study) focused on active engagement with patients and team members leading to more clarity in tasks and greater inclusivity in healthcare provision ^33^. Contributions of nurses and pharmacists (male or female) are often muted on ward rounds, excluding them from decision-making, leading to delays in key IPC and antibiotic activities. Critically, AMS programmes remain anchored in well-resourced health systems and institutions, or leading flagship academic centres ^34– 37^.

Findings from quantitative research studies describe existing disparities in healthcare access and antibiotic use. A study conducted in Europe reported that, in the community, women are 27% more likely to be prescribed an antibiotic than men, even after adjusting for urinary tract infections, which more commonly presented in women in the community ^38^. Gendered interactions have also influence antibiotic prescriptions, despite guidelines that stipulate optimised antibiotic prescribing. A study from the Netherlands identified female general practitioners prescribe less antibiotics than male counterparts, especially in consultations with female patients ^39^. In a study of over one billion patient visits in the USA, 11.3% included an antibiotic, with total and inappropriate antibiotic use highest amongst black (122.2 and 78.0 per 1000) and Hispanic patients (138.6 and 79.8 per 1000) ^40^. Nationally and globally we do not have robust data, consistently gathered for benchmarking and evaluation on antibiotic use and AMR by gender, race, ethnicity. Of the global databases for prevalence of key infections by syndrome and pathogen, including susceptibility, only one (WHO Global Antimicrobial Resistance and Use Surveillance System – GLASS) has data disaggregated by gender ^41^. There is a clear need for gender-disaggregated data, as well as class-, age- and ethnicity-disaggregated data.

Behaviours, roles and opportunities within the healthcare sector are mired within differentials of power ^42^. Patients and HCWs are not alone in confronting power differentials, and having to work within particular hierarchical frameworks. The families of patients must confront the same. Traditional, familial gender hierarchies determine whether and how women seek healthcare ^43^. Patients are rarely included in decision-making about their care, which is dissipated amongst a community of socially connected individuals; IPC and AMR policies which embrace these informal roles are urgently needed ^44^.

The COVID-19 pandemic has exacerbated the socioeconomic disparities that impact health with deprivation and ethnic marginalisation associated with worse outcomes ^45^. COVID-19 also led to the adoption of new behaviours, including improved hand hygiene and mask wearing which overlap with IPC behaviors needed to contain AMR ^46, 47^. The perceived risks, however, and the motivation to act upon these risks, are not aligned across the COVID-19 and AMR pandemics. IPC- and AMR-related health promotion efforts amongst HCWs need to recognise the influence of social networks and sociocultural perspectives. Our research from India and South Africa has demonstrated the missed opportunities in IPC, with nurses bearing the majority of IPC and antibiotic administration burden ^33, 48^, as well as the critical roles that carers ^49^, and patients ^50, 51^ have in the process of IPC and antibiotic access, use, and knowledge. This is despite the lack of engagement with these groups on these issues ^9, 24, 33, 52, 53^.

The primary question guiding the PROTEA study is: ‘How do power relations related to accountability and decision-making intersect with socio-cultural and economic drivers to shape healthcare access and health-providing behaviours of HCWs, patients and carers in relation to IPC and antibiotic use?

Secondary questions will explore: Is there a difference in quantity and quality of antibiotic prescriptions by gender of the prescriber/doctor? Does this vary by region (North versus South India and India versus South Africa). What is the effect of physician/surgeon and patient gender concordance on antibiotic prescriptions, length of stay and re-admission rate? What is the impact of gender composition of medical and surgical teams on surgical outcomes, length of stay and re-admission rate?

## Protocol

### Study design

**Figure 1.**
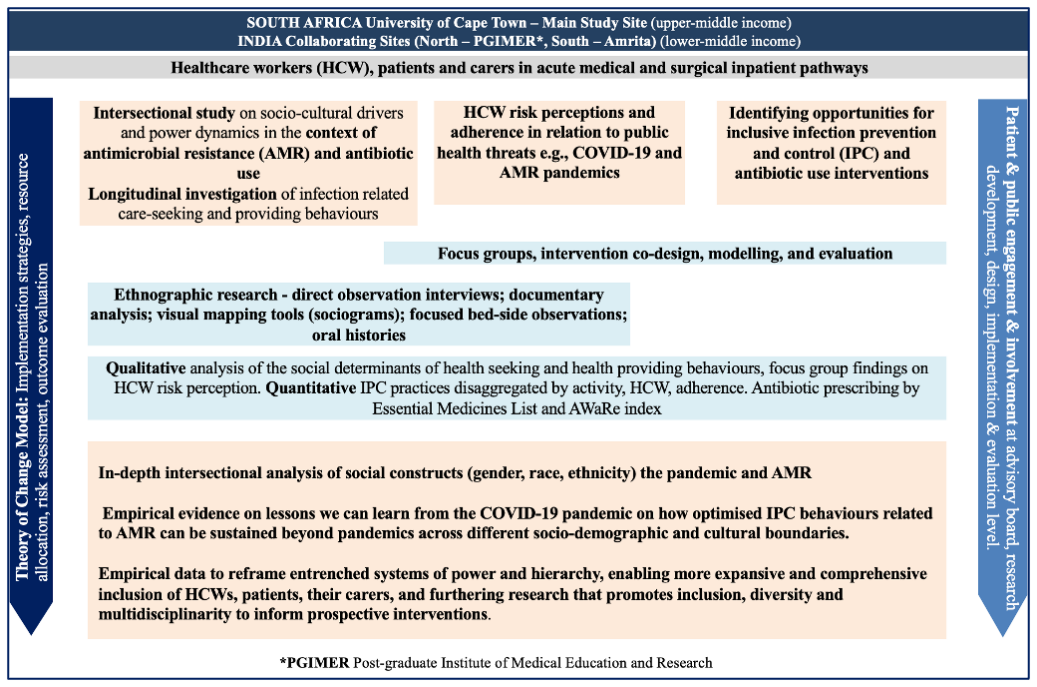
Overview of the Power Relations in Optimisation of Therapies and Equity in Access to Antibiotics (PROTEA) Study: India and South Africa.

The PROTEA study overview is provided in Figure 1. A scoping review of literature on how power manifests in healthcare systems across cultural and social boundaries and the implications of this for IPC practices and antibiotic use will be conducted.

The planned qualitative studies, including in-depth, open-ended interviews and observational research will investigate how social constructs (including race, gender, class) and resultant power dynamics intersect with IPC and AMR. It will examine how this knowledge can be leveraged to optimise practices. Using the narratives and experiences of individuals from different socio-demographic and professional backgrounds, the ways in which relative power, hierarchies and social constructs shape the experiences in IPC, AMS, and antibiotic use will be explored. This will lead to a better understanding of how existing structures, systems, and processes in healthcare disempower engagement and limit inclusion of marginalised people from different socioeconomic, cultural, and professional backgrounds. Focus groups and interviews will also investigate HCW risk perception in relation to IPC, antibiotic use and AMR. For observations in non-institutional setups such as those in the community, the observations would centre around availability and access to WaSH facilities. This is critical in defining how HCWs understand, and process risk associated with their practices in IPC, antibiotic use and AMR. Findings will provide lessons in sustaining the positive changes in IPC practices that have emerged from the COVID-19 pandemic and apply these to AMR interventions conducted as a component of this project.

### Study population

***Study sites*.** South Africa and India are upper- and lower- middle-income countries respectively. Between 2000 and 2010, 76% of global antibiotic consumption was attributable to the BRICS countries, with India as the largest consumer of antibiotics in human health ^54^. There is limited access to affordable healthcare with neither country achieving universal healthcare coverage. In South Africa, over 80% of access to healthcare is in the public sector ^55^. In India, access to the available free public health care is severely compromised due to high demand and limited resources at government facilities. This drives private sector access to health care to up to 65% of all access, with patients paying out-of-pocket ^56^. With some recent initiatives of Central and State Governments this expenditure has been curtailed to an extent. However, more needs to be done.

Groote Schuur Hospital is a 950-bed government-funded public hospital in Cape Town. Amrita Hospital, in Kerala South India is a not-for-profit 1350-bed private tertiary center. The study sites provide means-tested subsidised care, run established AMS programmes, and play key roles in AMS initiatives in their respective contexts. The Post-Graduate Institute of Medical Education and Research (PGIMER) in the state on Chandigarh in North India is a 1850-bed government, multi-specialty hospital serving the populations of five neighbouring states. The study sites were selected because, despite operating in health systems with limited resources, they have established strategies to optimise the use of antibiotics ^35, 36, 57^. Since the original application and with further funding from the WHO TDR, we have had the opportunity to expand the study to Himachal Pradesh, Haryana, and Bihar.

***Sample selection process*.** Through purposive sampling we will recruit: (1) HCWs involved in patient care in the included specialities, (2) patients (medically cognisant, and able to provide an account of their history), and (3) their carers, such as family members. Hospital managers involved in patient pathways will also be eligible to be included. Full informed written consent will be taken before participant recruitment. Sampling will ensure diversity in race, caste, class, gender, and roles. Local researchers from the team familiar with the local vernacular including isiXhosa, Afrikaans, Malayalam and Hindi will be able to conduct interviews for non-English speaking participants.

***Sample size calculation***

*Qualitative methods*

Ethnographic fieldwork will span six months in South Africa (eight weeks unstructured followed by 16 weeks structured data collection, providing 150–250 hours of observation), and six months (3 months per site) of intensive field work in India (four weeks unstructured followed by eight weeks of structured data collection, providing between 75–150 hours of observation, 50–70 anticipated interviews per country). Documentary analyses of policies, guidelines, and protocols together with composition of existing teams will be analysed to provide the macro healthcare organisation context.

Focus group sessions with HCWs, managers, and support staff will investigate risk perception and self-reported IPC and antibiotic use behaviours. There will be three focus groups per hospital per country (total 12 sessions, with ten participants in each session), with participants in each country stratified by professional group to maximise participation in the sessions. Topic guides and scenarios will be developed for each site and the sessions will be audio recorded and transcribed verbatim. A series of workshops (three per site) will be held to work with HCWs of different specialties and professions to explore their self-perceived and actual roles in IPC and AMR and how they navigate these roles in existing hegemonies.

*Quantitative methods*

Selected interventions will be co-developed with clinical teams and implemented. The bedside observation tool for IPC practices, in addition to indicators for IPC and antibiotic use and specific to selected interventions will be used to evaluate the outcomes. Over the span of 24 months, on general medicine and surgical wards in participating sites data will be gathered on quality of antibiotic use, gender concordance of prescribers and patients, and clinical outcomes including length of hospital stay, re-admission, antibiotic days. We anticipate 1000 in-patients per specialty per site to be recruited to this phase of the study.

### Outcome measures and analysis

***Reported experiences*.** Data from ethnographic research including focused bedside observations of IPC practices, visual mapping tools (sociograms), structured, and semi-structured interviews, and documentary analysis, will provide longitudinal qualitative (field notes, interview transcripts) data. Interviews and field notes will be transcribed verbatim and coded line-by-line using Nvivo. To compare the strength of analysis from Nvivo with manual qualitative analysis, qualitative data from one of the health sites will be coded manually without the use of a software programme. Documentary analyses of policies, guidelines, and protocols together with composition of existing teams will be analysed to provide the macro healthcare organisation context. Going beyond documenting the intersectionality of the social constructs and their inter-relationship with infection-related health, this research will deconstruct their mediating factors. The existing evidence on power and intersectionality in health will be used to investigate the interdependence of the social determinants of health and their impact on IPC, AMR, and AMS ^58, 59^. Descriptive analysis will be inductive using a grounded theory approach informed by: the investigators’ existing evidence from the field, reflexivity, and sensitising concepts from the literature on professional role identity, role boundaries, power and social constructs ^4, 26, 48, 60^. To frame this in the context of IPC and AMR, we will draw on our existing work describing the team dynamics, culture and hierarchies that influence care in hospitals ^24, 27, 28, 61^. Occupational consciousness will be applied as a framework to understand how and why dominant practices are sustained and how to disrupt these dominant narratives to diversify and transform roles in IPC and AMR ^23^.

Focus group data will provide an in-depth understanding of how HCWs’ perceived risks and motivation to respond to specific threats of the COVID-19 and the AMR pandemic are formed. The findings will be used to develop and test a toolkit for advocacy for leaders in AMR and healthcare to promote policy dialogue on this issue which will include need-based, co-designed and inclusive interventions. Through deliberation and co-design with HCWs we will provide access to enhanced evaluation tools for IPC for managers and policy makers.

***Observed metrics*.** Ethnographic research will comprise of bedside observations of IPC practices and antibiotic management (using a tool we have piloted), visual mapping tools (sociograms), interviews – face-to-face or via zoom, and documentary analysis to gather longitudinal qualitative data. Sociograms will capture and describe team dynamics, communication and tasks performed in relation to IPC and antibiotic use at the bedside. Discrete episodes of care related to IPC and antibiotic use will be analysed. Field notes from the observations will provide rich data on the cultural dynamics between participants. Using purposive sampling from the observations HCWs will be invited to participate in focus groups. Purposive sampling will assure ethnic, gender, and educational representation with participants in each stratified by professional group. Transformational leadership workshops will use the same sampling methods and be held in participating sites to test and validate a self-advocacy tool with patients and HCWs.

The face-to-face interviews will use a pre-piloted semi-structured interview guide. All study material are available as Extended Data ^62^. Adding context to the observational data, they provide the opportunity to confirm directly with the participants some of the assumptions made in the observations. Data analysis will be recursive and data will be collected until thematic saturation is achieved. The interviews will follow direct observations to ensure that participants are engaged about their attitudes and practices without affecting their behaviours during the observations. Interviews conducted in languages other than English will be transcribed before being translated to English, and back-translated again for accuracy.

***Quantitative data*.** The bedside observation tool for IPC practices, in addition to indicators for IPC and antibiotic use, and specific to selected interventions, will be used to evaluate the outcomes. Data gathered will include antibiotic use, categorised by national essential medicines list categories and WHO AWaRe index. Prescriptions will be analysed for evaluating patterns of antibiotic use according to predefined metrics. For assessment of appropriateness local applicable guidelines will be adopted ^63^. Proportion of prescriptions deemed rational and components (choice, dose, frequency, route, duration) of rational prescription will be summarised using descriptive statistics. IPC practices will be aggregated by activity e.g., WHO five moments of hand hygiene, missed opportunities, assessment of adherence to expected standards, tasks performed; and disaggregated by activity, profession and HCW demographics. Descriptive statistical analysis will be used to interpret the data.

The number of cases of bacterial AMR infections from patient populations in each site will be collected. Working with surgical and medical teams at participating sites, we will analyse which types of infection and antibiotic management data are collected during daily activities that could augment prediction of AMR outbreaks using innovative surveillance methods. The data will be disaggregated by socio-demographic factors, key risk factors, and outcomes mapped against the care journey, providing patient level data on AMR and related outcomes. While detailed socio-economic data are not generally available, and patient address data are often imperfect, we will pilot new methods for sustainable incorporation of this data in routine care. Where appropriate socioeconomic status of participants will be evaluated using existing indices developed for LMICs ^64, 65^. The main outcomes of interest will be related to appropriateness of antibiotics prescribed. Potential key predictors will be social and gender norms, education level, employment level, history of illnesses, health of the patient, and access to WASH. Bivariable and multivariable regression analyses will be used for each outcome of interest including socio-economic predictors of antibiotic prescription, and variables significantly associated with the outcomes at a p-value <0.05 will be entered into a multivariate regression model ^62^.

### Challenges and how these will be addressed

The breadth and scope of this research is structured for deliverability, across four years. The research team are well placed to deliver on the objectives, based on their track-record in both countries. Delays in identifying and developing tools, methodologies and interventions will be anticipated and flagged, timelines revised for deliverables working closely with sponsors and mentors.

The team have experience in multi-site international research, during the pandemic. Timelines on deliverables will be revised; qualitative interviews can be conducted via phones or other digital/online media in case of any future outbreak. Site work can be arranged with local researchers following appropriate IPC procedures. The role of researchers as outsiders or insiders to the context is a potential limitation. The team have extensive social science research in both settings. Biases will be addressed through reflexivity, methodological and analytical triangulation and working closely with research participants and collaborators. Triangulation, respondent validation and reflexivity will ensure the findings remain faithful to the participants’ experience. Methodological triangulation will be achieved through the observations, face-to-face interviews, and documentary analysis, as well as focus groups. Data triangulation will be achieved by collecting data over different periods of time, from different persons and different countries, hospitals, teams, and specialties. This approach of combining data from different sources and persons will help increase the validity and reliability of the data collected and will help to overcome intrinsic biases, including observer bias. The positionality of the researchers within the research will be consistently assessed and described through reflexivity among researchers in group discussions.

### Dissemination of outcomes

Through patient, public, and community engagement we will develop multiple formats of sharing the findings of this research including deliberation sessions with the local community, workshops, videos and animation and use of social media, writing of articles and blogs in local languages led by the early career researchers employed to the research team. The scientific communication of this research will be through academic and conference presentations of the research.

### Study status

We have begun a scoping review on the role of power in healthcare in relation to infection related practices. We have initiated a series of workshops in India and South Africa on the topic of gender, AMR, and climate as part of follow on WHO funding exploring narratives from healthcare workers, activists, lay members of the public, and policy makers. The process of ethical approval to study race, caste, gender from a sociocultural perspective in India and South Africa has taken one year, and there are many lessons in the process on how to study race and gender in these different settings. We are in the process of writing our experience as a practice piece for ethical review committees. We have recruited researchers to the studies in all sites and are in the process of recruiting clinical teams and sites. One of the comments from the Wellcome Trust review panel for this research was that in India, we had selected atypical sites that did not represent the sociocultural and economic diversity and challenges of the country. We have since, engaged with Society for Health information Systems Programme (HISP) India (co-author Arunima Sehgal Mukherjee) and through a series of workshops in the states of Himachal Pradesh, Haryana, and Bihar been able to identify sites where we can conduct this research in communities most affected by the burden of AMR. Similarly in South Africa we are working closely with Eh!woza (co-author Anastasia Koch) for sustainable meaningful community engagement of this research in the township of Khayelitsha in Cape Town and will be expanding the research to Khayelitsha District Hospital.

## Conclusions

**Figure 2.**
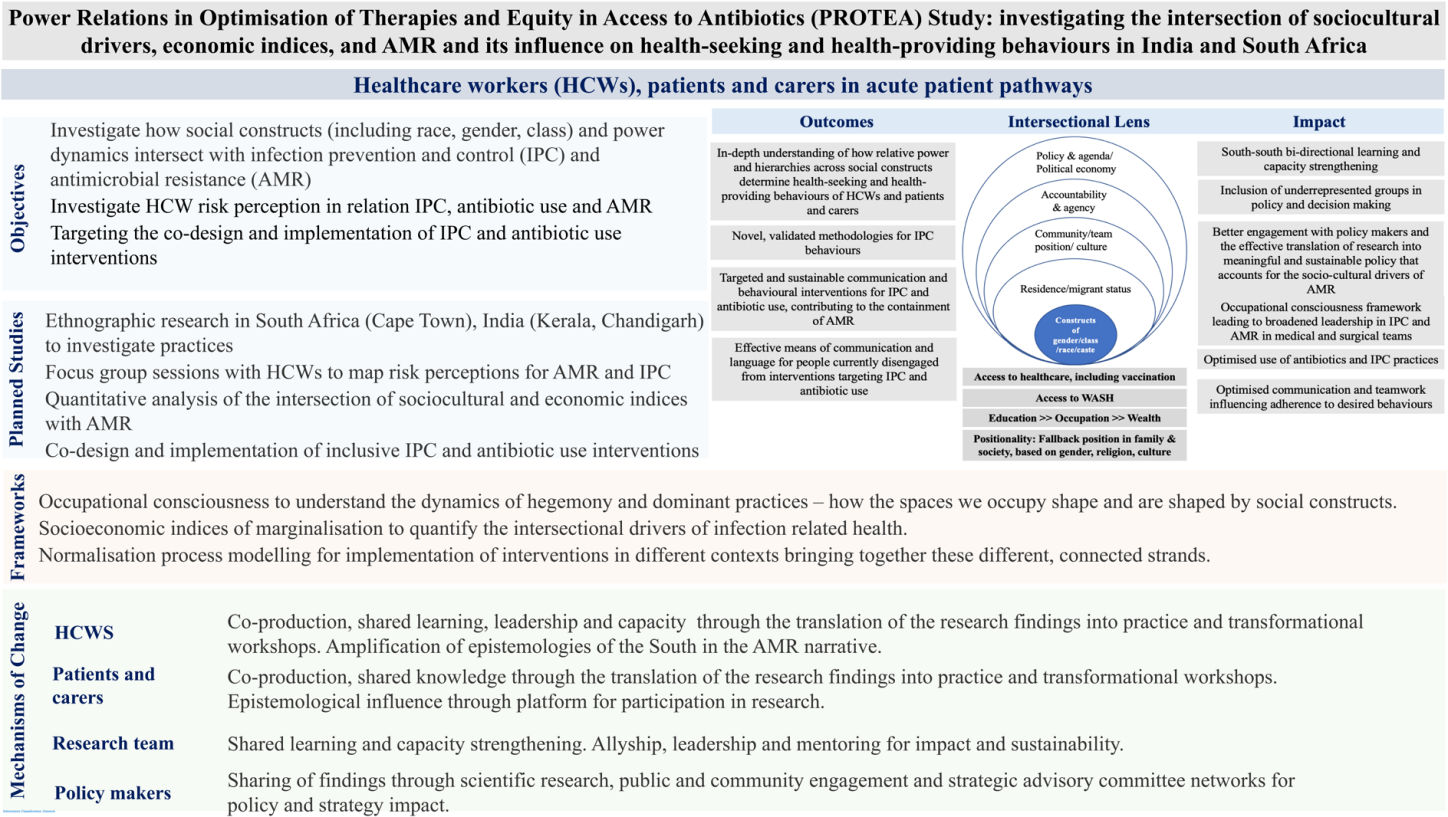
The proposed Theory of Change model for the PROTEA study.

This research is built on a decade of work, which has generated empirical knowledge in this field. Guided by a theory of change model ( Figure 2) the findings have relevance beyond IPC and AMR providing a mechanism for dismantling and reframing the structures which obstruct equitable access to health and healthcare, for those less privileged.

## Ethical statement

Ethical permission has been provided via the University of Cape Town (UCT) Human Research Ethics Committee [PROTEA Study HREC Reference 693/2023] in South Africa on May 22 2024. Local ethical approval has been granted in India as part of the CAMO-Net research programme grant from Amrita Vishwa Vidyapeetham Institute of Medical Sciences (ECASM-AIMS-2023-332). In the Post-Graduate Institute of Medical Education and Research the approval for this research has been granted under the CAMO-Net research programme IEC-09/2022-2567. These ethical review applications build upon existing research by the team at UCT: FWA00001637; IRB00001938 and in India (Amrita) IEC‐AIMS‐2018‐INF.CONT‐005A. We are applying for Indian Council of Medical Research for National Ethical Approval via Society for Health information Systems Programme (HISP) India. Study in each site will be conducted following requisite approvals being granted.

## Data Availability

No data are associated with this article. ZivaHub: Power Relations in Optimisation of Therapies and Equity in Access to Antibiotics (PROTEA) Study: investigating the intersection of sociocultural drivers, economic indices, and AMR and its influence on health-seeking and health-providing behaviours in India and South Africa, https://doi.org/10.25375/uct.26056177.v1 ^62^. This project contains the following extended data: Full protocol, including Participant Information Leaflets, Consent Forms, Interview Guides, Observations Guide, and Study Poster Data are available under the terms of the Creative Commons Attribution 4.0 International license (CC-BY 4.0).
