# Supplementary material for: Power Relations in Optimisation of Therapies and Equity in Access to Antibiotics (PROTEA) Study: investigating the intersection of socio-economic and cultural drivers on antimicrobial resistance (AMR) and its influence on healthcare access and health-providing behaviours in India and South Africa
Source: Wellcome Open Res. 2024 Jul 24;9:400. [Version 1] doi: 10.12688/wellcomeopenres.20193.1 (PMC11417457; doi:10.12688/wellcomeopenres.20193.1)
Supplement: Peer review history

# Reviewer report: Reviewer response for version 1

**DOI:** 10.21956/wellcomeopenres.22356.r96171

**Published:** 2024-09-22

**Authors:** Eva Maria Krockow

**Affiliations:** 1 The University of Leicester, Leicester, UK

The PROTEA study protocol presents an ambitious research plan to address socio-economic and cultural factors that may contribute to antimicrobial resistance in LMICs (India and South Africa). Using a variety of methods (including ethnography, focus groups and routine quantitative hospital data), the authors aim to identify and address power relations in antibiotic stewardship and infection control. The ultimate aim is to pave the way for equitable healthcare access and more appropriate use of antibiotics across diverse settings. I applaud the authors' mixed-methods approach, the focus on both medical and surgical settings and the nuanced considerations of socio-economic factors, including considerations of caste in India as well as potential language barriers. I only have a few suggestions for improvement at this stage. 

**General comments:**

- I would have liked to see more detail on the integration of qualitative and quantitative data. The authors provided a few statements around data triangulation in the Challenges section, but I think more could be said about how the different data sources will be combined to produce a holistic picture.
- There seems to be a mismatch between the number of participating hospital sites in India (5) and South Africa (1). I appreciate that India is a bigger country with a higher degree of regional variability, but I was wondering how this mismatch is justified? I also note that one of the Indian hospital sites is a private healthcare facility, while the other sites appear to be public hospitals? It might be worth commenting on the selection of hospitals and the effect of private versus public institutions.

**Minor comments:**

- Figure 1 could be adapted for additional clarity. For example, it is unclear to me what the red and blue colour-coding refers to. I also struggle to understand what individual text boxes represent (e.g. research studies, analytical approaches or outcomes).
- The Method lists study sites in Kerala, Chandigarh, Cape Town, Himachal Pradesh, Haryana, and Bihar. It further states that there will be three focus groups per hospital per country, with 12 sessions in total. If there are 6 sites, shouldn’t there be 18 focus groups?
- Please provide more justification for the following statement: To compare the strength of analysis from Nvivo with manual qualitative analysis, qualitative data from one of the health sites will be coded manually without the use of a software programme.
- In the context of the interview plans, the authors state that data will be collected until thematic saturation will be achieved. It might be worth adding considerations about information power (see Malterud et al., 2016) to guide the number of interviews.

Is the study design appropriate for the research question?

Yes

Is the rationale for, and objectives of, the study clearly described?

Yes

Are sufficient details of the methods provided to allow replication by others?

Yes

Are the datasets clearly presented in a useable and accessible format?

Not applicable

Reviewer Expertise:

Health-related decision making and application of social science methods in the context of antibiotic use and antimicrobial resistance

I confirm that I have read this submission and believe that I have an appropriate level of expertise to confirm that it is of an acceptable scientific standard.

## References

[1] : Sample Size in Qualitative Interview Studies: Guided by Information Power. *Qual Health Res* .2016;26(13) : 10.1177/1049732315617444 1753-1760 10.1177/1049732315617444 26613970

# Reviewer report: Reviewer response for version 1

**DOI:** 10.21956/wellcomeopenres.22356.r93709

**Published:** 2024-09-02

**Authors:** Tess Johnson

**Affiliations:** 1 Ethox Centre, University of Oxford, Oxford, England, UK

Please note that as a theoretical bioethicist by training, I cannot comment on details of the methodology used. My expertise lies with assessment of ethical questions relating to the work, and general review of the area and its contributions to the literature. Further peer review will be needed for adequate assessment of the methods.

 This paper is a study protocol, detailing a mixed methods study examining healthcare providers’, patients’ and carers’ perspectives on social, economic and structural drivers of AMR-associated behaviours in hospital settings including antibiotic prescription and use, infection prevention and control measures, and levels and forms of hospital care.

 The study can be anticipated to fill a gap in the literature, gathering data at study sites in an upper-middle income country (South Africa) and a lower-middle income country (India). The results can be expected to inform future stewardship and related policy measures, given the plan for policy engagement within the protocol.

 The rationale and objectives of the study are clearly described. The study is linked to previous work by members of the team, and it clearly builds appropriately on that work. However, the authors could do more to draw clear links between, on the one hand, inequities in decisional power, access to care, and likelihood of antibiotic prescription and use, and on the other hand, ethically relevant points such as injustices in healthcare systems, or morbidity and mortality associated with AMR, in order to further motivate and justify their study purpose. At a couple of points in the text there is also switching between inequity and inequality, which should be clarified.

 I cannot answer whether the study design is appropriate for the research question, nor whether the method is sufficiently detailed for replication.

 The study design is presented in a clear and accessible format, through Figure 1, and questions that one may be left with about practical impact of the work are addressed in the theory of change (Figure 2).

**A couple of general comments:**

- In the introduction, there are a couple of places where the authors could offer additional definitions, e.g. AMR first mention, structural and social determinants of health first mention. Neither are strictly necessary.
- In the abstract, a couple of points are repeated, primarily the note on study sites in the final paragraph on page 2.
- In the introduction (para 2 on page 3), it is unclear whether the study populations chosen will face reasons 1), 2), and 3) for being most affected by AMR, or whether they may face just one or two of these. The list needs an ‘and’ or an ‘or’ for clarity on this point.

Is the study design appropriate for the research question?

Yes

Is the rationale for, and objectives of, the study clearly described?

Yes

Are sufficient details of the methods provided to allow replication by others?

Partly

Are the datasets clearly presented in a useable and accessible format?

Not applicable

Reviewer Expertise:

bioethics (theoretical)

I confirm that I have read this submission and believe that I have an appropriate level of expertise to confirm that it is of an acceptable scientific standard.
